# Long-Term Outcome of Unprotected Left Main Percutaneous Coronary Interventions—An 8-Year Single-Tertiary-Care-Center Experience

**DOI:** 10.3390/jpm15070316

**Published:** 2025-07-15

**Authors:** Orsolya Nemeth, Tamas Ferenci, Tibor Szonyi, Sandor Szoke, Gabor Fulop, Tunde Pinter, Geza Fontos, Peter Andreka, Zsolt Piroth

**Affiliations:** 1 Gottsegen National Cardiovascular Center, 1096 Budapest, Hungary; orsolya.nemeth@gokvi.hu (O.N.); tibor.szonyi@gokvi.hu (T.S.); sandor.szoke@gokvi.hu (S.S.); gabor.fulop@gokvi.hu (G.F.); tunde.pinter@gokvi.hu (T.P.); geza.fontos@gokvi.hu (G.F.); peter.andreka@gokvi.hu (P.A.); 2 Semmelweis University Doctoral School, 1088 Budapest, Hungary; 3 Physiological Controls Research Center, Obuda University, 1034 Budapest, Hungary; ferenci.tamas@nik.uni-obuda.hu; 4 Department of Statistics, Corvinus University of Budapest, 1093 Budapest, Hungary

**Keywords:** left main coronary artery, percutaneous coronary intervention, outcome

## Abstract

**Background/Objectives**: Randomized studies of patients with unprotected left main coronary artery (ULMCA) disease involve highly selected populations. Therefore, we sought to investigate the 60-month event-free survival of consecutive patients undergoing ULMCA percutaneous coronary intervention (PCI) and determine the best risk score system and independent predictors of event-free survival. **Methods**: All patients who underwent ULMCA PCI at our center between 1 January 2007 and 31 December 2014 were included. The primary endpoint was the time to cardiac death, target lesion myocardial infarction, or target lesion revascularization (whichever came first) with a follow-up of 60 months. **Results**: A total of 513 patients (mean age 68 ± 12 years, 64% male, 157 elective, 356 acute) underwent ULMCA PCI. The 60-month incidence of events was 16.8% and 38.0% in elective and acute patients, respectively. There were significantly more events in the acute group during the first 6.5 months. Of the risk scores, the ACEF (AUC = 0.786) and SYNTAX II (AUC = 0.716) scores had the best predictive power in elective and acute patients, respectively. The SYNTAX score proved to be the least predictive in both groups (AUC = 0.638 and 0.614 in the elective and acute groups, respectively). Left ventricular function (hazard ratio (HR) for +10% 0.53 [95% CI, 0.38–0.75] and 0.81 [95% CI, 0.71–0.92] in elective and acute patients, respectively) and, in acute patients, access site (femoral vs. radial HR 1.76 [95% CI, 1.11–2.80]), hyperlipidemia (HR 0.58 [95% CI, 0.39–0.86]), and renal function (HR for +10 mL/min/1.73 m^2^ higher GFR: 0.87 [95% CI, 0.78–0.97]) were independent predictors of event-free survival. **Conclusions**: Acute ULMCA PCI patients have worse prognosis than elective patients, having more events during the first 6.5 months. Besides anatomical complexity, clinical and procedural parameters determine the prognosis.

## 1. Introduction

Significant unprotected left main coronary artery (ULMCA) stenosis without revascularization is associated with high morbidity and mortality due to the large amount of myocardium supplied by the diseased vessel. Early clinical studies proved that coronary artery bypass grafting (CABG) offered survival advantage over the then-standard medical therapy in patients with stable angina and ULMCA disease [1,2]. Thanks to the improvements in medical therapy and devices, most notably drug-eluting stents (DESs), percutaneous coronary intervention (PCI) has appeared as a good alternative to CABG in selected patients. Multiple randomized trials have compared the two revascularization strategies [3,4,5,6,7]. According to their results, PCI with DESs can be an acceptable alternative for selected ULMCA patients with low or intermediate anatomical complexity. According to the current European guidelines, CABG remains the gold-standard revascularization strategy for significant ULMCA stenosis [8]. However, these studies involve highly selected populations; therefore, their conclusions have limited generalizability. There are several clinical conditions (e.g., cardiogenic shock or severe comorbidities) where the surgical risk is unacceptably elevated, and PCI is the only revascularization option. We only have little “real-world” data describing the long-term outcome of non-selected populations undergoing ULMCA PCI. We therefore aimed to characterize the 5-year outcome of consecutive patients undergoing percutaneous ULMCA revascularization at our tertiary-care center between 1 January 2007 and 31 December 2014 and sought to determine the predictors of event-free survival.

## 2. Materials and Methods

### 2.1. Study Design and Population

In this study, we enrolled all patients who underwent ULMCA PCI at our tertiary-care center between 1 January 2007 and 31 December 2014. No exclusion criterion was applied. Baseline characteristics, coronary angiograms, and details of the PCI were collected from the institutional database. Follow-up data were obtained from the institutional database; in cases where sufficient information was not available, we contacted patients, their families, physicians, and regional hospitals by phone or e-mail, and the national death registry was reviewed. The cause of death was determined using discharge summaries or autopsy reports, if available. Before the intervention, all conscious patients gave written informed consent. In the case of unconscious patients, the intervention was indicated as a life-saving procedure. The study was approved by the Regional Institutional Scientific and Research Ethics Committee of Semmelweis University (SE RKEB number: 82/2021).

### 2.2. Procedure

In general, the choice of devices for the intervention was left to the operator’s discretion. DES implantation was recommended. Distal ULMCA lesions were treated with one or two stent techniques, using the provisional T-stenting technique as default, but the choice of stenting strategy was also at the operator’s discretion. During the study period, the availability of intravascular ultrasound (IVUS) and optical coherence tomography (OCT) was very limited. The use of intra-aortic balloon pump (IABP) and that of fractional flow reserve (FFR) were left to the operator’s discretion.

After PCI, guideline-directed medical therapy was recommended in all patients which included 100 mg of aspirin and 75 mg of clopidogrel daily for 12 months. Prasugrel and ticagrelor were licensed only during the study period. Unfractionated heparin (UFH) was used as the periprocedural anticoagulant therapy. The use of glycoprotein IIb/IIIa inhibitors (GPIIb/IIIa) was at the operator’s discretion, mainly as a bail-out. The dose of UFH was 100 IU/kg, and 50–70 IU/kg when in combination with a GPIIb/IIIa.

### 2.3. Study Definitions

Acute indication was defined as acute coronary syndrome and/or hemodynamic instability; acute patients were admitted to the catheterization laboratory within 24 h of hospital admission. Death was considered cardiac unless an unequivocal alternative cause was present. The Fourth Universal Definition of Myocardial Infarction (UDMI) was used to define myocardial infarction (MI) [9]. Target lesion revascularization (TLR) was defined as repeat percutaneous or surgical revascularization for at least 50% restenosis of the treated lesion and a 5 mm adjacent proximal or distal segment. In-stent restenosis was defined as an at least 50% narrowing in the previously successfully treated lesion detected by angiography. Stent thrombosis was defined according to the definition of the Academic Research Consortium [10]. Renal function was defined as a glomerular filtration rate (GFR) calculated by the Cockcroft–Gault formula. We used standardized scores (SYNTAX, SYNTAX II, additive and logistic EuroSCORE, EuroSCORE II, ACEF, GRACE 2.0 score) to determine the individual risk of patients.

### 2.4. Outcome and Follow-Up

The primary endpoint of the study was the time to the composite of cardiac death, target lesion MI (TLMI), and TLR, whichever came first, within 60 months. The follow-up period was 60 months. Angiography was not mandated during follow-up; it was left to the discretion of the attending physician.

### 2.5. Statistical Analysis

Continuous variables are presented as mean ± standard deviation, while categorical variables are presented as counts (percentage).

Given the competing risk situation (non-cardiac deaths are not considered events), cumulative incidence function was used to estimate mortality. As an exception, in the univariate comparison between acute and elective subjects, the time-varying hazard ratio was estimated in a Cox proportional hazards model using the scaled Schoenfeld residuals with smoothing using natural cubic splines [11].

Time to event was modeled in a multivariable manner using Fine and Gray competing risks regression [12]. All variables were entered without variable selection, and continuous variables were assumed to be linear. The multivariable model is described by hazard ratios (HRs) and 95% confidence intervals (CIs). Variables were considered significant if *p* < 0.05.

The predictive power of different scores was calculated using time-dependent receiver operating characteristic (ROC) curves and the area under them (AUC) with 60 months as the time point used as the primary endpoint. R statistical program package version 4.4.3 was used to carry out the calculations using package tidycmprsk version 1.1.0.

## 3. Results

### 3.1. Patients and Procedures

From 1 January 2007 to 31 December 2014, a total of 513 patients underwent ULMCA PCI at our center. Among these, 157 (30.6%) had elective, and 356 (69.4%) had acute ULMCA PCI. The baseline clinical, angiographic, and procedural characteristics are summarized in Table 1 and Table 2 and Supplemental Table S1. In the elective group, 15 patients refused CABG whereas 142 were contraindicated by the cardiac surgeon because of the comorbidities, coronary anatomy, or the age of the patient. Among the acute patients, 6 refused CABG whereas the vast majority (350 patients) were found to be unsuitable for CABG because of hemodynamic status, hyperacute presentation, coronary anatomy, comorbidities, or age. In all these cases, the only remaining revascularization option was PCI, which was felt to be a better treatment option than medical therapy alone.

The mean age of patients was 66.3 ± 10.7 years in the elective group and 69.2 ± 11.9 years in the acute group. The majority was male in both groups, and approximately 40% were diabetic. Echocardiography detected left ventricular ejection fraction (LVEF) before the intervention was lower in the acute group (41.3% ± 15.2) than in the elective group (55.8% ± 13.2). In the acute group, non-ST-segment elevation acute coronary syndrome was the indication in 196 (55.1%) cases, ST-elevation myocardial infarction in 94 (26.4%) cases, and cardiogenic shock caused by myocardial infarction in 66 (18.5%) cases.

The mean SYNTAX score was lower in the elective than in the acute group (21.1 ± 9.2 vs. 29.3 ± 12.4, respectively). Distal ULMCA PCI was performed in 133 (84.7%) patients in the elective group and 297 (83.4%) in the acute group. A true bifurcation ULMCA lesion was present in 35 (22.3%) elective, and 142 (39.9%) acute patients; among these, the most common type was the Medina 1,1,1 lesion in both groups.

IABP was used in 9 (5.7%) cases in the elective group and 128 (36.0%) patients in the acute group. Of note, during this period, IABP was the only available mechanical circulatory support device at our center. The use of FFR occurred mainly in the elective group (29.9%). The majority of the interventions were performed through the radial artery. Regarding time-related changes, most of the interventions were performed through the femoral artery between 2007 and 2009 (93–100%); thereafter, the use of the femoral artery was reduced (42% in 2010, 37% in 2011, 12% in 2012, 21% both in 2013 and 2014) with the radial artery becoming the main vascular access site. The use of intracoronary imaging during the study period was very limited (1.4%): in the study population, six IVUS and one OCT investigation were performed.

The default strategy in distal ULMCA PCI was the provisional T-stenting strategy. Of the two-stent strategies, the T-stenting technique was applied most frequently (10.8% in elective, and 15.7% in acute cases). Most of the interventions were performed using DESs; first-generation DESs were used in 21 (13.4%) elective and 64 (18.0%) acute cases, and new-generation DESs in 118 (75.2%) and 207 (58.1%) cases, respectively. Bare-metal stents (BMSs) were implanted mainly in acute patients, and their use declined over the years.

Complete revascularization was achieved during the index procedure in 22 (33.3%) patients with cardiogenic shock and 33 (35.1%) patients presenting with ST-elevation myocardial infarction (STEMI); another 1 (1.5%) and 4 (4.3%) had complete revascularization during the hospitalization period, respectively. Incomplete revascularization was mostly due to chronic total occlusions or lesions that were unsuitable for intervention because of poor run-off or small vessel diameter.

Guideline-recommended medical therapy was applied. A total of 470 (91.6%) patients surviving to discharge from our hospital received dual antiplatelet medication. More potent P2Y12 inhibitors were not available during this period, so all patients were on 100 mg of aspirin and 75 mg of clopidogrel; furthermore, 388 (82.6%) received beta blockers, 417 (88.7%) angiotensin-converting enzyme inhibitors or angiotensin receptor blockers, and 450 (95.7%) statins.

### 3.2. Follow-Up

The event-free survival status is known in 465 patients (90.6%). A total of 145 (31.2%) patients suffered at least one primary outcome event (cardiac death, TLMI, or TLR) within 60 months; this is shown in Supplemental Figure S1. During follow-up, one acute patient underwent heart transplantation after four months.

### 3.3. Outcome of Elective and Acute Patients

In-hospital mortality was 1.3% in the elective and 11.8% in the acute group. In the elective group, two patients died during the index hospitalization: one patient died on the seventh day because of the complication of another procedure (left atrial appendage closure), while the other patient developed hemodynamic instability at the end of the diagnostic coronary angiography, showing critical distal LM stenosis, and despite successful LM PCI and IABP, mechanical ventilation, and vasopressor therapy, they died on the next day due to critical hypotension.

Of the acute patients, 42 (11.8%) died during the index hospitalization; the cause was refractory heart failure in 31, electrical storm in 6, left ventricular free wall rupture in 1, sepsis in 3, and multiorgan failure in 1.

**Figure 1:**
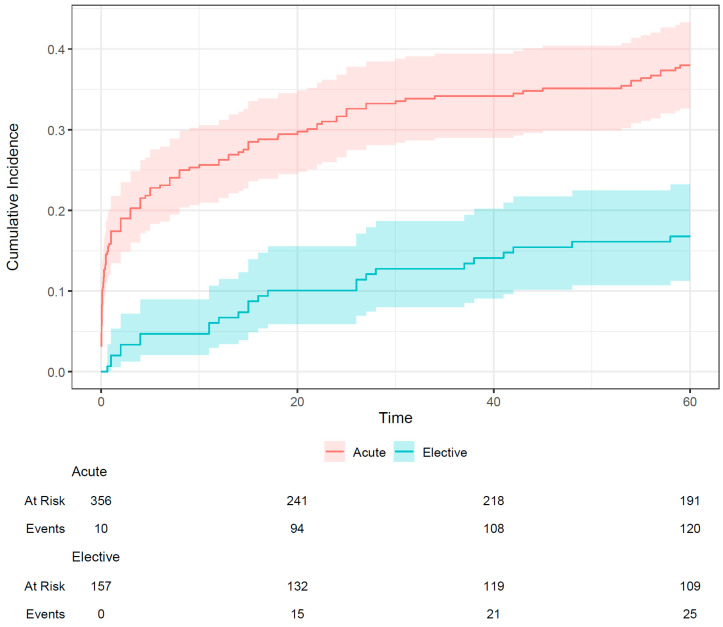
Incidence of the primary endpoint (cardiac death, target lesion myocardial infarction, target lesion revascularization) of elective and acute patients undergoing unprotected left main percutaneous coronary intervention. The shaded area indicates the 95% confidence interval.

The incidence of the primary endpoint at 60 months was higher in the acute group (38.0%) than in the elective group (16.8%); this is shown in Figure 1. A total of 52 (33.1%) elective patients died during follow-up; of these, 19 (12.1%) had a cardiac cause of death. In addition, 181 (50.8%) acute ULMCA PCI patients died within 60 months; of these, 95 (26.7%) had a cardiac cause. The incidence of non-cardiac death at 60 months was similar in both groups (15.4% and 14.2%, respectively).

In the elective group, 25 patients suffered at least one event during follow-up, including three TLMI and eight TLR. There were four other target vessel myocardial infarctions (TVMIs)—where the ULMCA was not involved—in the elective group which occurred after 2, 4, 6, and 49 months (Supplemental Table S2). Successful redo PCI was performed in all cases. In the acute group, 120 patients suffered at least one event during follow-up. Detailed follow-up data are shown in Table 3.

Of note, there was no definite or probable acute stent thrombosis in the elective group. Among the acute patients, none had definite and one had probable acute stent thrombosis. In the elective group, none had definite subacute and one patient had probable subacute stent thrombosis. Among acute patients, three had definite subacute stent thrombosis (two treated by successful redo PCI, while one died during reintervention), and twelve had possible subacute stent thrombosis. No late stent thrombosis was confirmed during the follow-up in either group.

During follow-up, significant ULMCA in-stent restenosis was detected in four elective patients, two of them associated with myocardial infarction in the 2nd and 15th month. ULMCA restenosis was detected in 28 acute patients, of which 12 were associated with myocardial infarction. All these patients were successfully treated by PCI or CABG. A total of 65 (41.4%) elective and 128 (36%) acute patients had recoronary angiography during the follow-up period; based on these, 23 and 79 revascularizations were indicated in the elective and acute groups, respectively. Of these, only nine asymptomatic patients without positive non-invasive tests underwent revascularization due to stenosis detected during routine recoronary angiography.

**Figure 2:**
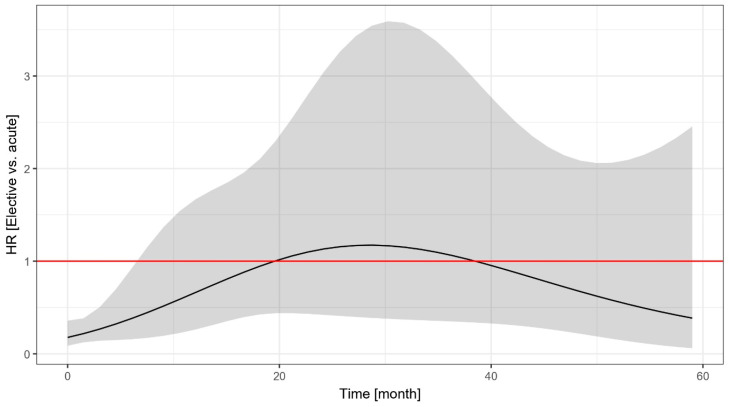
Time-dependent change in the hazard ratio of the event-free survival of elective vs. acute patients undergoing unprotected left main percutaneous coronary intervention. The shaded area indicates the 95% confidence interval. HR: hazard ratio.

As shown in Figure 2, the hazard of untoward events was higher among the acute patients than the elective patients, but this difference was significant only in the first 6.5 months, when most of the events occurred in the acute group. Due to the wide confidence intervals, the exact length of this period with increased risk (and the overall pattern of the time-varying hazard ratio) is difficult to judge.

### 3.4. Predictive Power of the Risk Score Systems

**Figure 3:**
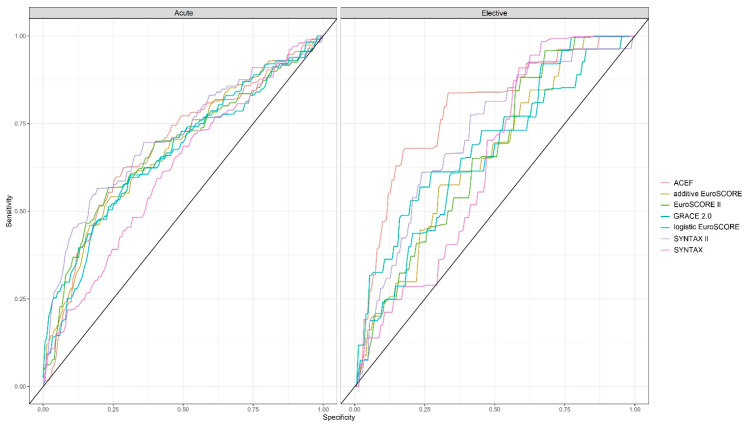
Power of the risk score systems to predict five-year event-free survival of acute and elective patients undergoing unprotected left main percutaneous coronary intervention.

The simple ACEF score (AUC = 0.786) and the SYNTAX II score (AUC = 0.717) were the best predictors of event-free survival at 60 months in the elective group, whereas the SYNTAX II score (AUC = 0.716) had the best predictive power in the acute group. The SYNTAX score, which only takes anatomical parameters into account, proved to be the least predictive in both the elective (AUC = 0.638) and the acute group (AUC = 0.614) (Figure 3). Supplemental Table S3 shows the AUC values of the risk score systems in acute and elective patients.

### 3.5. Predictors of Event-Free Survival

**Figure 4:**
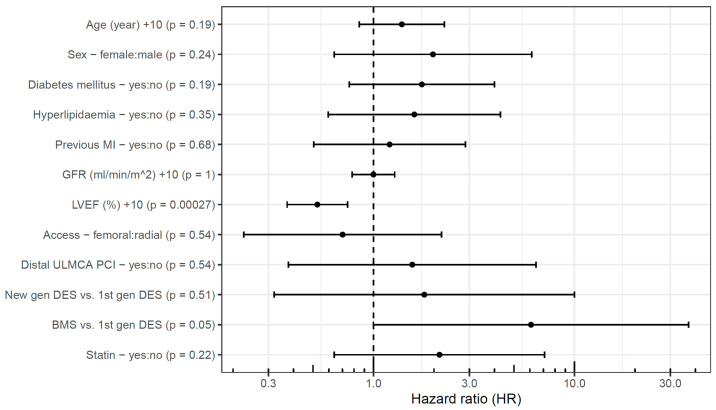
Predictors of event-free survival after elective unprotected left main percutaneous coronary intervention. BMS: bare-metal stent; DES: drug-eluting stent; gen: generation; GFR: glomerular filtration rate; LVEF: left ventricular ejection fraction; MI: myocardial infarction; PCI: percutaneous coronary intervention; ULMCA: unprotected left main coronary artery.

Independent predictors of event-free survival were investigated using multivariable analysis, separately in the two patient groups. LVEF emerged as a significant predictor in the elective patients (Figure 4). A 10%-higher LVEF (HR 0.53 [95% CI, 0.38–0.75]) was associated with a lower hazard of cardiac death, TLMI, or TLR, whereas older type of stents implanted in the ULMCA tended to be associated with a higher hazard, but the confidence interval is too wide and did not reach statistical significance.

**Figure 5:**
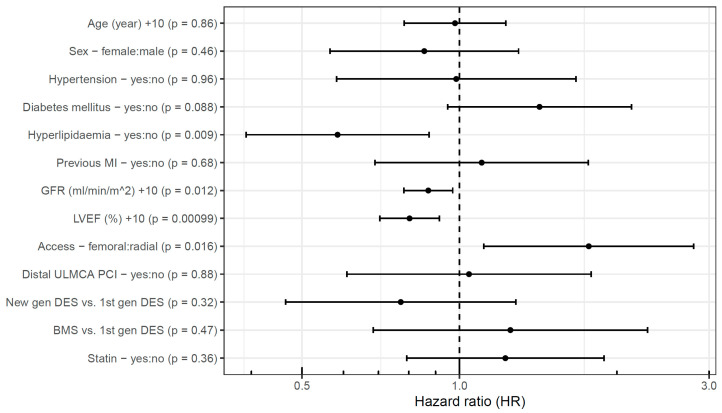
Predictors of event-free survival after acute unprotected left main percutaneous coronary intervention. BMS: bare-metal stent; DES: drug-eluting stent; gen: generation; GFR: glomerular filtration rate; LVEF: left ventricular ejection fraction; MI: myocardial infarction; PCI: percutaneous coronary intervention; ULMCA: unprotected left main coronary artery.

Similarly, in the acute group, a 10%-higher LVEF (HR 0.81 [95% CI, 0.71–0.92]) proved to be a significant predictor of event-free survival. Besides this, a 10 mL/min/1.73 m^2^ higher GFR (HR 0.87 [95% CI, 0.78–0.97]) was associated with a lower hazard of events, whereas femoral access (HR 1.76 [95% CI, 1.11–2.80]) was associated with a higher hazard of events in 60 months (Figure 5). Oddly, the presence of hyperlipidemia (HR 0.58 [95% CI, 0.39–0.86]) was associated with lower hazard of events. Statin use upon admission was not associated with the outcome, and even when including this variable in the model, the association of hyperlipidemia with the primary endpoint remained significant.

## 4. Discussion

In this study, we present the 5-year outcome of consecutive patients after ULMCA PCI performed between 2007 and 2014 at a single tertiary-care center. The salient findings of our study can be summarized as follows. First, in a population of patients who were unsuitable for or declined surgical revascularization, the 60-month event rates are high after ULMCA PCI, especially in acute patients within the first 6.5 months. Second, the SYNTAX score had the weakest power among the risk scores to predict untoward events. Third, LVEF and (in acute patients) renal function and vascular access site were independent predictors of 60-month event-free survival.

According to the randomized controlled trials, PCI can be an acceptable alternative revascularization strategy to the gold-standard CABG in ULMCA patients with low and intermediate anatomical complexity [3,4,5,6,7]. Their results showed that in the PCI arm, there were fewer periprocedural events, but in the long term, significantly more repeat revascularizations and late spontaneous myocardial infarctions occurred. The latest 2024 ESC Guidelines for the management of chronic coronary syndromes give a class IA recommendation for CABG in unprotected left main disease to improve survival and lower the risk of spontaneous myocardial infarction and repeat revascularization; however, in chronic coronary syndrome patients with significant left main coronary stenosis of low complexity in whom PCI can provide equivalent completeness of revascularization to that of CABG, PCI is recommended as an alternative to CABG, given its lower invasiveness and non-inferior survival [8].

ULMCA disease involves bifurcation in 80% and is frequently accompanied by downstream disease. The current practice of distal ULMCA PCI is to keep the procedure as simple as possible and limit the number of implanted stents. The default strategy of ULMCA bifurcation intervention when the side branch is not involved is the provisional T-stenting approach [13]. In the EBC-MAIN study, the provisional approach was associated with fewer target lesion revascularizations at 12 months and at 3 years [14,15]. In our study, the provisional T-stenting technique was the default strategy. However, as opposed to the EBC-MAIN study in which intravascular imaging was used in 40%, during the study period, the use of intracoronary imaging was very limited at our center; currently. the appropriate use of IVUS in ULMCA and complex PCIs has been proven to reduce long-term events [16,17].

In our cohort, incomplete revascularization was mostly due to chronic total occlusions or lesions that were unsuitable for intervention. According to a recent post hoc analysis of the extended PRECOMBAT study, there was no significant difference between PCI and CABG according to the completeness of revascularization [18]. Importantly, in the EXCEL trial, incomplete revascularization was associated with increased rates of death and cardiac events, especially untreated high-grade lesions in the circumflex artery [19].

Our report did not aim to compare the two revascularization strategies. Due to the consecutive nature of inclusion of patients not undergoing CABG for various reasons, the patient population is very high risk. The majority of the patients appeared unsuitable for CABG because of comorbidities, coronary anatomy, age, or hemodynamic status. Heart team decisions were followed, except when the patient refused to undergo CABG, in acute STEMI, and in patients with cardiogenic shock.

In our study, mortality and event rates were high: the 60-month survival of elective patients was 67.8%, while that of acute patients was 47.8%. A cardiac cause of death was found in 12.1% and 26.7% in elective and acute patients, respectively, which means that the cause of death was cardiac in one in four elective patients dying within 5 years, but in the acute group, almost half of the total mortality was cardiac. However, despite all efforts being made to establish the exact cause of death, the retrospective nature of our study is a limitation to this analysis.

Elective patients had a 16.8% incidence of target lesion failure (cardiac death, TLMI, or TLR) within 5 years, whereas acute patients had a 38.0% incidence. Non-cardiac death did not differ between the two groups. In our analysis, two-thirds of the TLRs occurred in the first two years, this was also shown in a study from four observational registries [20]. The target event curves of elective and acute patients separated from each other right at the beginning. We found that the outcome of acute patients is worse, related to more events mainly during the first 6.5 months. This suggests that the first 6.5 months are critical in acute ULMCA patients. Of note, based on the current European guidelines, chronic coronary syndrome is considered 12 months after an acute coronary syndrome event [21]. The acute ULMCA group was defined as having acute coronary syndrome and/or hemodynamic instability; although stable non-ST-segment elevation acute coronary syndrome patients differ from those with cardiogenic shock, by definition, all acute patients were admitted to the catheterization laboratory within 24 h of hospital admission, and in our cohort, percutaneous coronary intervention was decided following surgical turndown. In addition, coronary artery disease is customarily grouped as chronic or acute coronary syndrome, and practice guidelines apply this distinction.

A recent meta-analysis of four randomized trials [22] showed that diabetes mellitus was associated with higher 5-year mortality, myocardial infarction, and repeat revascularization rates in patients with ULMCA disease; cardiovascular mortality was higher after PCI in diabetic patients with a high SYNTAX score. In our study, taking clinical parameters into consideration resulted in significant improvements in event prediction. Among the risk scores, the SYNTAX score, which is currently the basis of decision-making [8], had the weakest predictive power for the 60-month event-free survival in both indication categories; all the other scores were similar and better. The SYNTAX II score proved to be the best in acute ULMCA PCI patients. In a prospective registry of patients with significant ULMCA or multivessel disease who were ineligible for CABG, the EuroSCORE II (and STS score) proved to be better predictors than the most contemporary NCDR CathPCI bedside risk model; however, surgical scores overestimated the short-term mortality [23]. In elective patients, the simple ACEF score, which takes only three (albeit very strong) predictive parameters (age, renal function, LVEF) into account, proved to be the best predictor. A recent meta-analysis of randomized trials showed that SYNTAX score should be used with caution to identify the most appropriate revascularization strategy [24]. Our ULMCA PCI analysis concurs with this.

The occurrence of events was proportionally more frequent with the decrease in the LVEF. Reduced renal function was also associated with poor outcomes, especially in acute patients. These parameters are known risk factors and are incorporated in several risk score systems. Kosmidouet al. [25] showed that these parameters were predictors of readmission after ULMCA revascularization, and readmission was independently associated with subsequent all-cause mortality and cardiovascular mortality.

Hyperlipidemia appeared as a protective factor against events in acute patients. Shrivastava AK et al. [26] confirmed a significant reduction in serum cholesterol level and elevation in triglyceride levels alongside other pro- and anti-inflammatory markers in the first two days of MI. Hyperlipidemia was diagnosed based on lipid levels measured on admission to our hospital and/or the presence of previous antilipemic therapy. Since our center is a national referral hospital, many patients arrived long after the onset of symptoms, thereby potentially reducing the lipid levels measured upon arrival. On the other hand, previous statin use is known to be protective against ischemic events, not to mention better compliance with the prescribed drug regime being a selection bias, again, confounding the correlation of hyperlipidemia diagnosis and prognosis. However, we found that statin use upon admission was not associated with the outcome, and even when including this variable in the model, the association of hyperlipidemia with the primary endpoint remained significant.

Previous practice included routine follow-up angiography after ULMCA PCI, but currently, recoronary angiography is unnecessary in asymptomatic patients with negative non-invasive tests [27,28]. In our study population, during follow-up, only 8.8% of the indicated revascularizations were in asymptomatic patients without positive non-invasive tests.

In our practice, after 2009, most of the interventions were performed through the radial artery. ULMCA PCI via the femoral as compared to the radial artery was associated with more events, with the difference reaching statistical significance in the acute group; however, this may be partly due to selection bias with hemodynamically unstable or highly complex patients being preferentially treated via femoral access. Today, the guideline-recommended access site for PCI is the radial artery [29].

The use of BMSs declined sharply; in fact, these were implanted in patients with a short expected survival time, mostly early on in the study period. In line with our findings, a meta-analysis showed that new-generation DES implantation was associated with a reduction in MI and all-cause and cardiac death [30]. In our analysis, less events occurred after ULMCA PCI with new-generation DESs, both in elective and acute patients; however, this difference did not reach statistical significance.

### Limitations

The study has several limitations. First, a relatively small number of patients were included. However, the consecutive nature of our study precludes selection bias. On the other hand, the studied patient population cannot be considered homogeneous; this is why the acute and elective groups were studied separately. Second, complete 60-month follow-up data were available in only 90.6% of the patients; however, follow-up was 100% complete in terms of cardiac death. Due to retrospective data collection, it was not always possible to clearly determine the cause of death, and non-fatal events may have been missed. Another limitation is the single-center nature of the study. During the study period, no mechanical circulatory support devices (e.g., Impella, TandemHeart) were available other than the IABP, intravascular imaging modalities were seldom applied, and some drugs (e.g., prasugrel, ticagrelor) were not yet used, let alone tailored antiplatelet therapy, in these high-risk patients. Importantly, interventional techniques may not represent those of today. The lack of routine angiographic follow-up may have resulted in some of the ULMCA in-stent restenoses not being detected and treated; however, given the resultant large amount of ischemic myocardium, this is unlikely to have changed the major findings. The lack of a comparator arm with surgical revascularization or medical therapy alone makes it difficult to contextualize whether our observed outcomes are acceptable. However, our analysis focuses on patients who had no alternative therapy given the clinical conditions (in acute and in some elective cases) of the patients or their adamant refusal of surgical revascularization (in elective cases). In patients presenting with acute coronary syndrome and significant left main stenosis, it felt completely unethical to withhold all forms of revascularization; therefore, medical-only therapy was not feasible and could not be analyzed. Indeed, we are not aware of any randomized clinical trial comparing left main revascularization modalities with each other and medical therapy in the literature. Access to non-invasive risk-stratification methods, like cardiac magnetic resonance imaging, was limited during the study period [31].

## 5. Conclusions

Our study shows that in terms of event-free survival, acute ULMCA PCI patients have a worse prognosis than elective patients, mainly related to significantly more events early after PCI. In our analysis with a limited number of patients and follow-up, the SYNTAX score was found to have the weakest power to predict 5-year untoward events. Beside anatomical complexity, clinical parameters are significant predictors of event-free survival. Careful planning of the intervention and the use of appropriate tools can improve the outcome in this high-risk population.

## Figures and Tables

**Table 1:** Baseline characteristics of the patients (n = 513).

	Elective (N = 157)	Acute (N = 356)
Age, year	66.3 ± 10.7	69.2 ± 11.9
Male sex	107 (68.2)	220 (61.8)
Hypertension	136 (86.6)	283 (79.5)
Diabetes mellitus	65 (41.4)	138 (38.8)
Treated with oral antidiabetics	45 (28.7)	90 (25.3)
Treated with insulin	20 (12.7)	48 (13.5)
Hyperlipidemia	112 (71.3)	236 (66.3)
Previous myocardial infarction	55 (35.0)	92 (25.8)
Previous PCI	46 (29.3)	60 (16.9)
Previous CABG *	7 (4.5)	6 (1.7)
GFR, mL/min/1.73 m^2^	68.8 ± 25.3	60.6 ± 25.2
Left ventricular ejection fraction, %	55.8 ± 13.2	41.3 ± 15.2
Clinical presentation		
Stable angina	157 (100)	n.a.
Non-ST-segment acute coronary syndrome	n.a.	196 (55.1)
ST-elevation myocardial infarction	n.a.	94 (26.4)
Cardiogenic shock caused by myocardial infarction	n.a.	66 (18.5)
SYNTAX score	21.1 ± 9.2	29.3 ± 12.4
Low (<23)	105 (66.9)	123 (34.6)
Intermediate (23 to 32)	35 (22.3)	92 (25.8)
High (>32)	17 (10.8)	141 (39.6)
SYNTAX II score PCI	34.5 ± 13.1	46.1 ± 16.4
SYNTAX II score CABG	34.1 ± 14.1	39.7 ± 28.4
EuroSCORE II	2.9 ± 4.0	17.4 ± 18.1
Additive EuroSCORE	4.3 ± 3.1	11.3 ± 4.1
Logistic EuroSCORE	5.2 ± 6.9	29.3 ± 22.3
ACEF	1.4 ± 0.6	2.0 ± 1.0
GRACE 2.0 score	92.5 ± 26.2	140.5 ± 38.9

Continuous variables are expressed as a mean ± standard deviation; categorical variables are expressed as numbers (percentages). CABG: coronary artery bypass grafting; GFR: glomerular filtration rate; PCI: percutaneous coronary intervention. * None of these patients had any patent graft to the left coronary system.

**Table 2:** Lesion and procedural characteristics (n = 513).

	Elective (N = 157)	Acute (N = 356)
Right dominant coronary artery	138 (87.9)	307 (86.2)
Occluded dominant RCA	14 (8.9)	78 (21.9)
True bifurcation	35 (22.3)	142 (39.9)
Trifurcation	18 (11.5)	59 (16.6)
Ostial/shaft ULMCA PCI	24 (15.3)	59 (16.6)
Distal ULMCA PCI	133 (84.7)	297 (83.4)
Provisional T-stenting	105 (66.9)	228 (64.0)
Final kissing balloon dilatation	116 (73.9)	222 (62.4)
Direct stenting	36 (22.9)	47 (13.2)
ULMCA PCI only	75 (47.8)	145 (40.7)
BMS	9 (5.7)	76 (21.3)
DES		
First generation	28 (17.8)	92 (25.8)
New generation	120 (76.4)	188 (52.9)
>1 stent implanted to the ULMCA	17 (10.8)	39 (11.0)
ULMCA stent diameter, mm	3.5 ± 0.3	3.4 ± 0.7
ULMCA total stent length, mm	22.6 ± 11.1	21.7 ± 10.0
Post-dilatation	150 (95.6)	321 (90.2)
Maximum post-dilatation pressure, atm.	18.3 ± 5.1	19.0 ± 6.7
Intra-aortic ballon pump	9 (5.7)	128 (36.0)
GPIIb/IIIa usage	2 (1.3)	35 (9.8)
Radial access	108 (68.8)	184 (51.7)
Use of FFR	47 (29.9)	13 (3.7)

Continuous variables are expressed as a mean ± standard deviation; categorical variables are expressed as numbers (percentages). BMS: bare-metal stent; DES: drug-eluting stent; FFR: fractional flow reserve; GPIIb/IIIa: glycoprotein IIb/IIIa inhibitor; PCI: percutaneous coronary intervention; RCA: right coronary artery; ULMCA: unprotected left main coronary artery.

**Table 3:** The 5-year outcomes (n = 465) *.

Outcome	Elective (N = 149)	Acute (N = 316)
Primary endpoint **	25 (16.8)	120 (38)
Cardiac death	19 (12.8)	95 (30.1)
TLMI	3 (2)	15 (4.7)
TLR	8 (5.4)	33 (10.4)
Target lesion PCI	7 (4.7)	26 (8.2)
Target lesion CABG	1 (0.7)	7 (2.2)
Definite stent thrombosis	0 (0)	3 (0.9)
Probable stent thrombosis	1 (0.7)	13 (4.1)

Continuous variables are expressed as a mean ± standard deviation; categorical variables are expressed as numbers (percentages). CABG: coronary artery bypass grafting; PCI: percutaneous coronary intervention; TLMI: target lesion myocardial infarction; TLR: target lesion revascularization. * Of the 513 patients, all follow-up data are available for 465. ** Composite of cardiac death, target lesion myocardial infarction, or target lesion revascularization within 60 months.

## Data Availability

The data that support the findings of this study are available from the corresponding author upon reasonable request.

## Supplementary Materials

The following supporting information can be downloaded at: https://www.mdpi.com/article/10.3390/jpm15070316/s1, Figure S1: Incidence of non-cardiac death and the primary endpoint (cardiac death, target lesion myocardial infarction, target lesion revascularization) of patients undergoing unprotected left main percutaneous coronary intervention. The shaded area indicates the 95% confidence interval; Table S1: Lesion and procedural characteristics (n = 513); Table S2: 5-year outcomes (n = 465) *; Table S3. AUC values of the risk score systems in acute and elective patients.

## Abbreviations

The following abbreviations are used in this manuscript:

AUC	area under curve
BMS	Bare-metal stent
CABG	coronary artery bypass grafting
CI	confidence interval
DES	drug-eluting stent
FFR	fractional flow reserve
GFR	glomerular filtration rate
GPIIb/IIIa	glycoprotein IIb/IIIa inhibitor
HR	hazard ratio
IABP	intra-aortic balloon pump
IVUS	intravascular ultrasound
LVEF	left ventricular ejection fraction
MI	myocardial infarction
OCT	optical coherence tomography
PCI	percutaneous coronary intervention
ROC	receiver operating characteristic
STEMI	ST-elevation myocardial infarction
TLMI	target lesion myocardial infarction
TLR	target lesion revascularization
TVMI	target vessel myocardial infarction
UDMI	Universal Definition of Myocardial Infarction
UFH	unfractionated heparin
ULMCA	unprotected left main coronary artery
