# Supplementary material for: Long-Term Outcome of Unprotected Left Main Percutaneous Coronary Interventions—An 8-Year Single-Tertiary-Care-Center Experience
Source: J Pers Med. 2025 Jul 15;15(7):316. doi: 10.3390/jpm15070316 (PMC12296052; doi:10.3390/jpm15070316)
Supplement: Supplementary file 1 [file jpm-15-00316-s001.zip › jpm-3623221-supplementary.pdf]

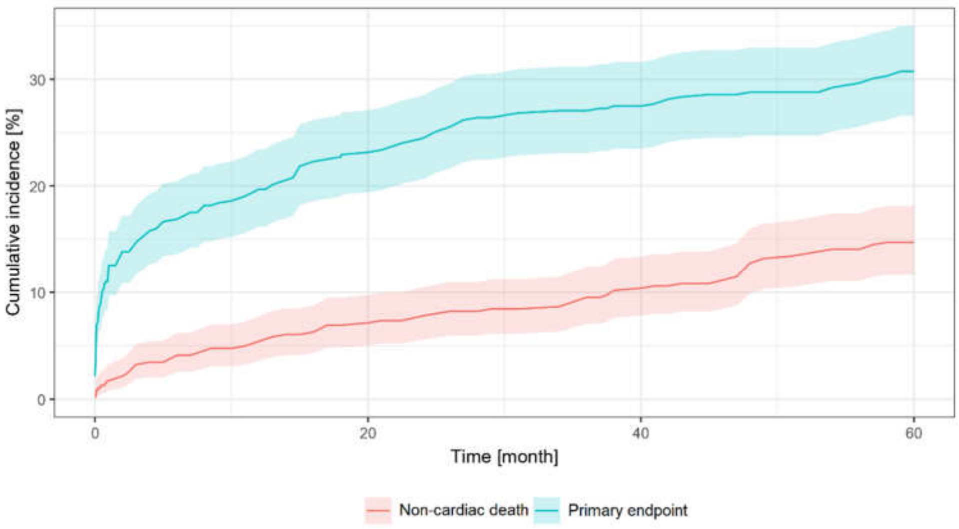

Figure S1: Incidence of non-cardiac death and the primary endpoint (cardiac death, target lesion myocardial infarction, target lesion revascularization) of patients undergoing unprotected left main percutaneous coronary intervention. The shaded area indicates the 95% confidence interval.

Table S1. Lesion and procedural characteristics (n=513)

	Elective (N=157)	Acute (N=356)
True bifurcation	35 (22.3)	142 (39.9)
Medina 1,1,1	23 (14.6)	106 (29.8)
Medina 1,0,1	2 (1.3)	23 (6.5)
Medina 0,1,1	7 (4.5)	13 (3.7)
Stenting strategy in distal ULMCA PCI		
Provisional T-stenting	105 (66.9)	228 (64.0)
T-stenting	17 (10.8)	56 (15.7)
Culotte	2 (1.3)	5 (1.4)
V-stenting	0 (0)	1 (0.3)
Mini-crush	0 (0)	1 (0.3)
ULMCA + 1 other vessel PCI	71 (45.2)	158 (44.4)
ULMCA + 2 other vessel PCI	11 (7.0)	49 (13.8)
ULMCA + 3 other vessel PCI	0 (0)	3 (0.8)
ULMCA POBA only	0 (0)	1 (0.3)
Brachial access	0 (0)	4 (1.1)
Femoral access	49 (31.2)	168 (47.2)

Categorical variables are expressed as numbers (percentages). PCI: percutaneous coronary intervention, POBA: plain old balloon angioplasty, ULMCA: unprotected left main coronary artery

Table S2. 5-year outcomes (n=465) *

**Outcome**	**Elective (N=149)**	**Acute (N=316)**
All-cause death	52 (34.9)	181 (57.3)
MI	14 (9.4)	41 (13)

TVMI	7 (4.7)	29 (9.2)
Revascularization in total	23 (15.4)	79 (25)
PCI	20 (13.4)	64 (20.3)
CABG	3 (2)	15 (4.7)
Target vessel revascularization	19 (12.8)	65 (20.6)

Continuous variables are expressed as a mean ± standard deviation, categorical variables are expressed as numbers (percentages). CABG: coronary artery bypass grafting, MI: myocardial infarction, PCI: percutaneous coronary intervention, TVMI: target vessel myocardial infarction

* Of the 513 patients, all follow-up data are available in 465

Table S3. AUC values of the risk score systems in acute and elective patients

	ACEF	EUROScore.II	GRACE.2	SYNTAX II PCI	SYNTAX	additive EUROScore	logistic EUROScore
Acute patients	0.6826 (0.6171-0.7583)	0.6821 (0.6185-0.7536)	0.6714 (0.608-0.736)	0.716 (0.6651-0.7771)	0.6142 (0.5546-0.6652)	0.6751 (0.616-0.7472)	0.666 (0.6107-0.7372)
Elective patients	0.786 (0.7006-0.8996)	0.6575 (0.5608-0.7684)	0.6893 (0.5623-0.8357)	0.717 (0.6033-0.8253)	0.6377 (0.5411-0.7346)	0.654 (0.5291-0.7566)	0.6565 (0.5588-0.7627)
